# Blood product usage and factors associated with transfusions in cats with hemoperitoneum: 33 cases (2018–2022)

**DOI:** 10.3389/fvets.2023.1204864

**Published:** 2023-07-13

**Authors:** Nicole Bunnell, April Blong, Debosmita Kundu, Jonathan Paul Mochel, Rebecca Walton

**Affiliations:** ^1^Las Vegas Veterinary Specialty Center, Las Vegas, NV, United States; ^2^Iowa State University, Ames, IA, United States; ^3^VCA West Los Angeles Animal Hospital, Los Angeles, CA, United States

**Keywords:** hemoperitoneum, hemorrhage, point-of-care diagnostics, transfusion therapy

## Abstract

**Objective:**

To evaluate blood product usage in cats with hemoperitoneum. To secondarily evaluate factors associated with transfusion administration and the outcome of cats with hemoperitoneum.

**Design:**

Retrospective study between the years 2018–2022.

**Setting:**

University veterinary teaching hospital and private practice hospital.

**Animals:**

33 cats admitted to the hospital diagnosed with hemoperitoneum from January 2018 to September 2022.

**Measurements and main results:**

Medical records were retrospectively reviewed; signalment, point-of-care diagnostics, effusion characteristics, and transfusion administration information was recorded. The most common etiology associated with hemoperitoneum was neoplasia (51.5%). Fifty-one percent (51.5%) of cats received a blood transfusion during hospitalization with the majority of cats receiving multiple transfusion types (69%). The etiology of hemoperitoneum was not associated with receiving a transfusion (*p* = 0.28) Point-of-care diagnostics including packed cell volume (PCV), total solids (TS) and platelet count were not significantly associated with receiving a transfusion (*p* = 0.317, *p* = 0.11 and *p* = 0.82, respectively). The PCV and TS of the effusion was also not significantly associated with transfusions (*p* = 0.91 and *p* = 0.63, respectively). Sixteen cats (48%) survived to discharge. Transfusions were significantly associated with outcome and cats that received a transfusion were more likely to survive to discharge (*p* = 0.008).

**Conclusion:**

In conclusion, hemoperitoneum from a variety of etiologies in cats is associated with a high proportion of transfusions. None of the evaluated point-of-care diagnostics were associated with transfusion administration in this study. Cats that received a transfusion were more likely to survive to discharge.

## Introduction

Hemoperitoneum is an uncommon but life-threatening emergency in cats with an incidence of up to 0.3% of cats over a 7-year period at a tertiary referral center (1). Hemoperitoneum results from a variety of traumatic and non-traumatic etiologies with the most common non-traumatic causes including neoplasia, coagulopathies, and hepatic necrosis (2–4). While the incidence of neoplastic etiologies is lower in cats than dogs, the prognosis of feline hemoperitoneum is still poor with a reported survival rate of 12% in a previous study (2).

Hemodynamic instability is common in feline hemoperitoneum secondary to hemorrhage and the decision to administer blood products is often subjective and based on clinician discretion. Transfusion triggers are often based on a multiple factors including cardiovascular parameters, such as heart rate, patient clinicial signs and the rate and magnitude of blood loss (5). Often, point-of-care diagnostics including PCV, TS and lactate are used to aid the decision of transfusion administration (6). In a recent study of canine spontaneous hemoperitonum, 47% of cases received packed red blood cell transfusions, however, no studies have evaluated blood product administration in feline hemoperitoneum (7). The purpose of this study was to determine the blood product usage and survival to discharge in cats with hemoperitoneum. Secondary objectives were to evaluate any associations between point-of-care diagnostics including PCV, TS, platelet count and PCV/TS of the effusion and transfusion administration. We hypothesized that feline hemoperitoneum would be associated with a high blood product usage and have a high mortality rate.

## Materials and methods

### Case selection

The computerized medical records database at a University veterinary teaching hospital and a large private practice hospital were searched for cats diagnosed with hemoperitoneum from January 2018 to September 2022. Computerized records were searched based on a diagnosis of hemoperitoneum, hemoabdomen or hemorrhagic abdominal effusion. Additionally, computerized records were search for financial codes for diagnostic abdominocentesis. Cats were included if diagnosed with a hemoperitoneum and hospitalized for a minimum of 12 h. A diagnosis of hemoperitoneum was made based on abdominal effusion noted on point of care ultrasound and a peritoneal fluid analysis documenting a PCV > 10%. Patients were excluded if they had a previous history of anemia within the prior 6 months to avoid affecting evaluation of point-of-care parameters, hemoperitoneum was not confirmed or they were euthanized prior to any treatment. Data collected included: patient signalment, peripheral PCV and TS at time of admission, PCV and TS of effusion, platelet count, survival to discharge, and whether the patient received a blood product during their hospitalization. Cats may have received intravenous fluids for stabilization prior to point-of-care data collection. In the authors institutions the PCV is obtained by the centrifugation of relevant samples in microhematocrit tubes and reading the percentage of packed cells using a microhematocrit reader. Total solids protein is determined using a refractometer. Automated platelet count is obtained as part of a complete blood count using an in-hospital hematology analyzer and platelet count was not confirmed with manual count. Transfusions recorded included packed red blood cells, fresh frozen plasma, and fresh whole blood.

### Statistics

The association between all variables of interest was evaluated with blood product usage. A multivariate logistic regression model was employed to assess the relationship between transfusion administration and various clinicopathologic variables. The dataset was analyzed to determine the proportion of missing data for independent variables. For descriptive statistics, the frequencies and percentages were used for reporting categorical variables. For continuous variables, descriptive statistics were reported as median followed by interquartile range (IQR). For all analyzes, statistical significance was considered when estimated *p*-values were < 0.05. All statistical analyzes were performed using R version 4.2.2 (A language and environment for statistical computing, R Foundation for Statistical Computing, Vienna, Austria).

## Results

A total of 45 cats diagnosed with hemoperitoneum were identified through the computerized search. Thirty-three cases met the inclusion criteria, after 12 cases were excluded due to lack of data recorded on presentation (3) or euthanasia prior to any treatment (8). Eighteen cats were spayed female, 14 were castrated males and one cat was an intact female. The median age of cats was 9 years (IQR 7.4 years) and the median weight was 4 kg (IQR 1.8 kg). Reported breeds included domestic short hair (23), followed by Siamese (4), domestic long hair (3) and one each of Ragdoll, Maine Coon, and Balinese.

In the majority of cases (17 cats, 51.5%), hemoperitoneum occurred secondary to neoplasia or suspected neoplasia. Five cats were definitively diagnosed with lymphoma based on fine needle aspirates, one cat was diagnosed with carcinomatosis based on fine needle aspirates, one cat with a biliary cystadenoma based on histopathology and 10 cats were suspected to have neoplasia due to a noted abdominal mass on abdominal imaging including ultrasound or CT scan. Seven cats had iatrogenic causes of hemoperitoneum, including five secondary to ovariohysterectomy and two due to a cystocentesis. Five were due to liver disease including hepatic fracture secondary to lipidosis (2), amyloidosis (1) or cholangiohepatitis (2). Two cats had underlying coagulopathy, one cat was thrombocytopenic due to bone marrow suppression from estrogen toxicity and one cat had prolonged coagulation times (prothrombin time and partial thromboplastin time) with no definitive cause identified, however, anticoagulant rodenticide was not suspected based on values and historical questioning. One cat had a peritoneal-pericardial diaphragmatic hernia and another cat experienced trauma causing hemorrhage. The cause of the hemoperitoneum was not significantly associated with transfusion (*p* = 0.28).

Twenty-seven cats had a PCV and TS value recorded at the time of presentation. The median peripheral PCV on presentation was 20% [IQR 10%, Reference range: 31–48%, (9)] and median peripheral TS on presentation was 6 g/dL [IQR 1.6 g/dL, Reference range: 6.1–8.0 g/dL, (8)] (Table 1). Eighteen cats had a platelet count performed on presentation; the median platelet count was 126 × 10^3^/uL [IQR 198 × 10^3^/uL, Reference range: 300–800 × 10^3^/uL, (8)]. Neither peripheral PCV (*p* = 0.317), TS (*p* = 0.11), or platelet count (*p* = 0.82) on presentation were significantly associated with receiving a transfusion. The median PCV of the abdominal effusion was 20% (IQR 13%). The PCV and TS of the effusion was also not significantly associated with transfusion (*p* = 0.91 and *p* = 0.63, respectively).

**Table 1:** Presenting point of care diagnostic results compared between cats with hemoperitoneum that did and did not receive a transfusion.

	Transfusion	No transfusion	*p*-value
Peripheral PCV %	20 (10)	23 (8)	0.32
Peripheral TS g/dL	6 (72.4)	7 (1.6)	0.12
PCV effusion %	20 (14.5)	20 (12)	0.91
TS effusion g/dL	4.8 (1.4)	5.3 (3.4)	0.63
Plt count × 10^3^/uL	126 (174)	135 (123)	0.82
Survival	12/16	4/17	0.008

Data are presented as median values and interquartile range is reported in parathensis.

A total of seventeen cats (51.5%) did not survive to discharge. Of those 17 cats that did not survive to discharge, 15 were euthanized (88%) and two died (12%). Of the non-survivors, one cat had a peritoneal-pericardial diaphragmatic hernia, l one cat had hepatic lipidosis and 15 were diagnosed with neoplasia. Based on retrospective evaluation of the record, twelve cats (80%) were noted to be euthanized due to a poor prognosis, 1 cat (6%) due to finances and reason for euthanasia was not noted in 4 cases.

Seventeen cats received a blood transfusion while 16 did not; overall 51.5% of cats received a blood transfusion. Sixteen cats received packed red blood cells, 10 received fresh frozen plasm and 2 received fresh whole blood. All cats were blood typed prior to transfusion and given type specific blood. All cats in this study were Type A and crossmatches were not performed in all cases. The median dose for cats that received blood products was as follows: packed red blood cells was 11.8 mL/kg (6.1–21.7 mL/kg), fresh frozen plasma 13 mL/kg (10.2–15.9 mL/kg), and fresh whole blood 22 mL/kg (19.2–26.4 mL/kg). Eight cats received a transfusion during initial stabilization, 2 in the pre-operative period, 2 during hospitalization and 2 in the intraoperative period and in 3 cats the timing of transfusion was unknown. Eleven of the 17 cats that received a transfusion (69%) received multiple transfusion types. Nine of the cats that received multiple transfusions received packed red blood cells and fresh frozen plasma, one cat received packed red blood cells and fresh whole blood and one cat received all three product types. Twelve of the cats that received a transfusion (71%) survived to discharge while only three of the 16 (31%) cats that did not receive a transfusion survived to discharge, cats that received a transfusion were significant more likely to survive to discharge (*p* = 0.008). A total of nine cats underwent an exploratory laparotomy for the diagnosed hemoperitoneum. All cats that underwent surgery received a transfusion. Three of the cats that underwent surgery had a hemoperitoneum secondary to postoperative hemorrhage from ovariohysterectomy, three cats for primary neoplasia, two cats for liver disease and one cat for a peritoneal-pericardial diaphragmatic hernia. Seven cats (78%) that underwent surgery survived to discharge.

## Discussion

The findings of this study were consistent with our hypothesis that cats with hemoperitoneum have a high mortality rate and high blood product usage (51.5%). Blood product administration was common in cats with hemoperitoneum, which is similar to previously reported rates of transfusions in dogs at 47% (7, 10). The majority of cats in this study had hemoperitoneum diagnosed seconday to neoplasia (51.5%), which is similar to previous studies (2). The most commonly diagnosed neoplasia was lymphoma (29%), however, 10 cats (59%) did not have a definitive cancer noted, which is in contrast to previous studies documenting hemangiosarcoma as the most common neoplasm diagnosed (2). This population of cats had a large proportion of iatrogenic hemoperitoneum (21%), with two of those occurring secondary to cystocentesis. Hemoabdomen secondary to cystocentesis has been reported in cats treated for urethral obstruction, however, iatrogenic hemoabdomen is not commonly reported in the literature (11). The etiology of the of hemoperitoneum was not associated with receiving a transfusion (*p* = 0.28).

Contrary to our hypothesis, point-of-care diagnostics were not associated with transfusion administration in this study. Transfusion triggers are poorly described and often debated in veterinary medicine; therefore, transfusion administration is often dictated based on clinical evaluation and physical exam findings, however, point-of-care diagnostics and their association with transfusion administration have been evaluated. Point-of-care diagnostics may be difficult in acute hemorrhage, such as hemoperitoneum, as the PCV will not drop until hours after hemorrhage due to the combination of splenic contraction and fluid shifts (12). When evaluated in dogs diagnosed with a hemoperitoneum, lower PCV, TS and platelet counts have been associated with an increased likelihood of transfusion administration (10). In this study, we aimed to evaluate if point-of-care bloodwork parameters in cats with hemoperitoneum were associated with transfusion administration. All cats in this study were anemic at the time of presentation with a median peripheral PCV of 20%; however, in contrast to previous studies in dogs, no association between peripheral PCV, TS, or platelet count and transfusions were noted (10). A significant difference may not have been noted in this population of cats as all cats were anemic on presentation. While point-of-care diagnostics did not correlate with transfusion administration in this study, it is very likely that these point-of-diagnostics did affect clinician decision to administer blood products and further prospective research is needed to evaluate these parameters and their correlation with transfusion administration.

In addition to point-of-care bloodwork, abdominal effusion scoring has been evaluated in dogs with blunt trauma and higher fluid scores were associated with a higher incidence of transfusions (13). Due to the retrospective nature of this study, abdominal fluid scores were not consistently noted or evaluated; however, abdominal effusion PCV and TS were evaluated. Abdominal effusion PCV and TS have not previously been evaluated regarding association with transfusion in hemoperitoneum in cats. In this study, the PCV and TS of the abdominal effusion was not associated with receiving a transfusion. In dogs, this has not been associated with receiving a transfusion, but trends can indicate active bleeding, which may necessitate a transfusion sooner (5). This may be due to the timeframe of hemorrhage or volume of abdominal hemorrhage in this population, however, more studies are needed to further evaluation the impact of abdominal effusion volume and diagnostics on transfusion need.

Transfusion administration was significantly associated with a better outcome and cats that received a transfusion had a higher survival (71 vs. 31%). The retrospective nature of this study precludes determination of specific transfusion triggers as the decision for transfusion administration was made at the attending clinician’s clinical discretion. Previous studies have documented blood product administration early in the resuscitative efforts of hemorrhagic shock is associated with improved survival due to improved tissue oxygenation, which may explain why cats that received a transfusion were more likely to survive to discharge (14). It is additionally possible cats that received a transfusion may been more likely to receive recommended treatment than those that did not receive transfusions, which may also explain the increased survival in this patient population.

Limitations of this study include its retrospective nature and the relatively low number of cases that were available for data analysis. Additionally, other factors, such as finances and prognosois likely affected the decision to treat and transfuse these cats. As more cats that received a transfusion survived to discharge, it is likely that these cats were more likely to be treated, therefore, finances likely played a large role in these findings. Point-of-care diagnostics were evaluated in regards to their impact on transfusion administration, in this study, platelet count was determined by complete blood cell count, which may be inaccurate in cats. Additionally, not all cats had point-of-care and complete testing, including coagulation panels, performed. Finally, the majority of cats that did not survive to discharge were euthanized.

In conclusion, about half of cats with hemoperitoneum received blood products. None of the presenting point-of-care diagnostics or effusion characteristics were associated with blood product administration. Overall, the prognosis for cats with hemoperitoneum is guarded with 48% of cats surviving to discharge, although cats that received a transfusion were significantly more likely to survive to discharge (71%).

## Data availability statement

The raw data supporting the conclusions of this article will be made available by the authors, without undue reservation.

## Author contributions

NB, AB, and RW contributed to study design. NB, AB, DK, JM, and RW contributed to data analysis and manuscript preparation. All authors contributed to the article and approved the submitted version.

## Conflict of interest

The authors declare that the research was conducted in the absence of any commercial or financial relationships that could be construed as a potential conflict of interest.

## Publisher’s note

All claims expressed in this article are solely those of the authors and do not necessarily represent those of their affiliated organizations, or those of the publisher, the editors and the reviewers. Any product that may be evaluated in this article, or claim that may be made by its manufacturer, is not guaranteed or endorsed by the publisher.

## Abbreviations

IQR: Interquartile range
PCV: packed cell volume
TS: total solids.
